# Light!

**Published:** 1871-07

**Authors:** P. H. Hayes


### LIGHT!

BY P. H. HAYES, M. D.

I take it few people are aware of the fact, that if the tadpole of the country mudpuddle—polliwog, the boys call him—be kept in a dark place, he can never turn into a frog. Dr. Hammond confined one of these frog babies in a dark box for 125 days, and he underwent no change, but on letting in the light he became a frog in 15 days. Now, herein is a proverb, for if the baby frog can't make the red blood in the dark which is to grow him to froghood, how can children, vastly above him in organization, without light, make the red blood they need to grow them to a vigorous manhood or womanhood.

I entered the house of my city cousin, John, in the middle of the day; his living rooms were all on the north side of his house, and so dark that on entering I could not clearly see anything. But after a little, as I talked with the wife and sported with the children, I saw how pale they were, and I said—Cousin John, you are my astute friend; you are an astronomer, and help the other astronomers, I suppose, to keep the solar system in order; but the human system, John, of this wife and these children, here's a lesson for you to read. What sort of blood do these pale ones have? It's blood and water mixed—Orange County milk, after it has been to the pump. Such a blood has little vital heat in it, so the pale ones must dress the more; they take cold easily, and they will have that nameless sort of spiritlessness, especially in the morning, which compels them to wind up on tea or coffee or other stimulant, before they can start for the day. Cousin John, if your wife cries easily, is hysteric-like, and complains of palpitation, you may be sure she has not got scripture blood in her arteries; I mean the kind which Moses said is the "life of the flesh."—You see, John, the brain can't give out in kind only as it receives; give it pale, watery blood, and weak and fitful is its work. So common is the pale-blood disease that doctors are forever giving tonics, nervines and blood restoratives—indeed, these are pretty much the backbone of the Materia Medica just now, and their names run in the doctors' heads so much that they can write quinine and iron as well in their sleep as when awake.

John, disease is just as natural—not *normal*—as health. Natural causes, I mean, determine the one as they do the other. What we call *nature* is just as law-working and law-abiding in giving us our diseases as our health. What is going on in your little family that you are all so pale? You are breaking organic laws of health. No, not *breaking*, no man ever did or ever can *break* a natural law; wherein they are transgressed they *break him*, and thus is it, that men become "utterly broken down." *Nature* will give us health or disease, cure or kill us, as we compel her.

Open your Shakespeare to "The Merchant of Venice," and see how the Prince of Morocco presses his suit on the quality of his blood:

"Mislike me not for my complexion,
The *shadowed livery* of the *burnished sun*;
Bring me the fairest creature northward born,
And let us make incision for your love
To prove whose *blood* is *reddest* his or mine."

Want of light pales and weakens every living thing, and since the wonderful revelations of the spectroscope, it is a question, *sub judice*, whether the blood does not absorb the "vapors of iron" from the sun's atmosphere.

Neuralgia, a disease of growing frequency, of which the single element is *pain*, is in nearly all of its forms but an outcry of the nerves for better arterial blood; and of all the chronic diseases which make so much work for the doctors, nine-tenths are from poor blood and poor nutrition.

John, I fancy I hear you say to your wife, "Do spunk up, Lizzie!" You might as well tell her to spunk up and conquer typhus fever as the pale-blood disease. Talk as we please about intrinsic powers of the will, there is no such thing. We are at the mercy of physical conditions, and the true qualification to be made in this statement is that we have largely the power to create or to choose our physical conditions, and therefore become responsible for their influences upon ourselves.

There is a mysterious power of nutrition for our finer nature in the light of the sun. Children at their play, are like birds in the trees, or lambs in the meadows, half their joy is in the sunny air. How does the sunlight in the summer waken myriads of insect life; it is as wine to them, the more they have the more they are stimulated to life and activity. They will not go where it is dark, so we darken our parlors to keep out the flies and the millers.

Florence Nightingale says it is the unqualified result of all her experience with the sick, that second only to their need of fresh air, is their need of light; that after a *close* room, what hurts them most is a *dark* room, and that it is not only light but direct sunlight that should come into every chamber.

People, she adds, think the effect is upon the spirits only. By no means. We must admit that light has quite as real and tangible effects upon the body. But this is not all. Who has not observed the purifying effect of sunlight upon the air of a room. It is a curious thing to observe how almost all patients will lie with their faces, as if by instinct, turned toward the light.

A densely shaded house is almost certain to be an unhealthy house. Darkness and dampness breed thousands of microscopic plants, creating mould, mildew and blasting, corrupting the air and filling it with the virus of fevers and many obscure diseases. Sunlight is a perfect caustic to all this poisonous vegetation, which can only flourish in secret places, darkness and dampness. All healthy and higher forms of plant life seek and enjoy the light.

Every lady who keeps house plants knows how they love the light and stretch their branches and turn their leaves toward the sunlight, and so she must turn them around often or they will grow one-sided.

There is not a man, woman or child that can have a high degree of health, who does not see and feel and enjoy sun and sky and out-door air daily.

"Truly, the light is sweet, and a pleasant thing it is to behold the light of the sun." We do not much wonder that in all ages, so many nations have worshipped the sun. They must have well observed what science now so abundantly proves, that it is the foundation of all earthly life and energy. And how sublimely beautiful and thrilling is that fiat of Jehovah, when he said, "Let there be light, and there was light;" or yet more sublime for brevity, the literal Hebrew: "Be light! and light was."

